# Antibiotic susceptibility of *Atopobium vaginae*

**DOI:** 10.1186/1471-2334-6-51

**Published:** 2006-03-16

**Authors:** Ellen De Backer, Rita Verhelst, Hans Verstraelen, Geert Claeys, Gerda Verschraegen, Marleen Temmerman, Mario Vaneechoutte

**Affiliations:** 1 Department of Clinical Chemistry, Microbiology & Immunology, Ghent University Hospital, UGent, Ghent, Belgium; 2 Department of Obstetrics & Gynaecology, Ghent University Hospital, UGent, Ghent, Belgium

## Abstract

**Background:**

Previous studies have indicated that a recently described anaerobic bacterium, *Atopobium vaginae *is associated with bacterial vaginosis (BV). Thus far the four isolates of this fastidious micro-organism were found to be highly resistant to metronidazole and susceptible for clindamycin, two antibiotics preferred for the treatment of BV.

**Methods:**

Nine strains of *Atopobium vaginae*, four strains of *Gardnerella vaginalis*, two strains of *Lactobacillus iners *and one strain each of *Bifidobacterium breve*, *B. longum, L. crispatus, L. gasseri *and *L. jensenii *were tested against 15 antimicrobial agents using the Etest.

**Results:**

All nine strains of *A. vaginae *were highly resistant to nalidixic acid and colistin while being inhibited by low concentrations of clindamycin (range: < 0.016 μg/ml), rifampicin (< 0.002 μg/ml), azithromycin (< 0.016 – 0.32 μg/ml), penicillin (0.008 – 0.25 μg/ml), ampicillin (< 0.016 – 0.94 μg/ml), ciprofloxacin (0.023 – 0.25 μg/ml) and linezolid (0.016 – 0.125 μg/ml). We found a variable susceptibility for metronidazole, ranging from 2 to more than 256 μg/ml. The four *G. vaginalis *strains were also susceptible for clindamycin (< 0.016 – 0.047 μg/ml) and three strains were susceptible to less than 1 μg/ml of metronidazole. All lactobacilli were resistant to metronidazole (> 256 μg/ml) but susceptible to clindamycin (0.023 – 0.125 μg/ml).

**Conclusion:**

Clindamycin has higher activity against *G. vaginalis *and *A. vaginae *than metronidazole, but not all *A. vaginae *isolates are metronidazole resistant, as seemed to be a straightforward conclusion from previous studies on a more limited number of strains.

## Background

Bacterial vaginosis is considered a common vaginal disorder in women of reproductive age. Whereas normal vaginal microflora consists of lactobacilli, especially *L. crispatus *[1-4], the disturbed vaginal microflora is characterized by the overgrowth of *Gardnerella vaginalis *and anaerobic bacteria such as *Mobiluncus *spp., *Mycoplasma hominis *and *Prevotella *spp. Recently several research groups showed – by means of cloning of the 16S rRNA-gene [3,5], by Terminal Restriction Fragment Length Polymorphism analysis of the 16S rRNA gene (T-RFLP) [6,7], by specific PCR [5,8,9], by Denaturing Gradient Gel Electrophoresis of the 16S rRNA gene (DGGE) [10] and by FISH [3,11] – that a previously unrecognized organism, *Atopobium vaginae*, was strongly associated with bacterial vaginosis and with *Gardnerella vaginalis*.

During the last decade, the interest for bacterial vaginosis increased because of reports of adverse sequelae of this disorder, such as preterm birth [12-14], pelvic inflammatory disease [15,16] and postpartum endometritis [17]. In addition, several publications showed that an altered vaginal microflora is linked to an increased susceptibility to the acquisition of HIV [18,19] and other sexually transmitted infectious agents such as *Neisseria gonorrhoeae *and *Chlamydia trachomatis *[18,20].

The severity of the consequences of such sequelae asks for an adequate treatment of bacterial vaginosis. Currently the preferred antibiotic treatment regimen consists of clindamycin or metronidazole (oral or intravaginal). Recurrence rates of up to 30% within 3 months after treatment have been reported [21,22]. This recurrence might be due to the survival of metronidazole or clindamycin resistant bacteria in the vagina, although Beigi *et al*. [23] showed recently that less than one percent of the cultivable vaginal anaerobic bacteria is resistant to metronidazole.

Another possible explanation might be the presence of some fastidious to unculturable, metronidazole resistant organism, a role for which *A. vaginae *is a likely candidate, since Geißdörfer *et al*. [24] and Ferris *et al*. [10] reported an MIC of > 32 μg/ml for metronidazole for the four isolates that were tested.

Here, we studied the susceptibility of nine other *A. vaginae *isolates for 15 antimicrobial agents by using the Etest and for comparison we included a limited number of other important vaginal bacteria such as lactobacilli, *G. vaginalis *and bifidobacteria and we compared our results with those of previously published articles.

## Methods

Nine strains of *Atopobium vaginae*, four strains of *Gardnerella vaginalis*, two strains of *Lactobacillus iners *and one strain each of *Bifidobacterium breve*, *B. longum, Lactobacillus crispatus, L. gasseri *and *L. jensenii *were tested against 15 antibiotics (ampicillin, azithromycin, bacitracin, cefuroxime, ciprofloxacin, clindamycin, colistin, doxycycline, kanamycin, linezolid, metronidazole, nalidixic acid, penicillin, rifampicin and vancomycin) [see Additional file 1]. Six strains of *A. vaginae *(CCUG 42099, CCUG 44116, CCUG 44258, CCUG 38953^T^, CCUG 44125, CCUG 44061) were kindly provided by the Culture Collection of Göteborg (Sweden). The other strains were obtained from studies by cultivating vaginal samples (with informed consent) and identified by tDNA-PCR and 16S rRNA gene sequencing [4].

Strains were cultivated anaerobically using the GasPak anaerobic envelope system (Becton Dickinson, Erembodegem, Belgium) at 37°C on Trypticase Soy Agar (TSA) + 5 % sheep blood (Becton Dickinson). Epsilometer tests (Etest AB Biodisk, Solna, Sweden) were used to determine the minimal inhibitory concentrations (MIC). An inoculum taken from TSA + 5% sheep blood was suspended in 0.5 ml of physiological water and adjusted to the turbidity of a 1 McFarland standard. Before inoculating the plates and testing for metronidazole, plates were anaerobically incubated during 24 h. After application of the epsilometer, test plates were anaerobically incubated at 37°C. The inhibitory concentration was defined as the value on the test strip scale at which the inhibition zone intersected the strip edge, according to the manufacturers' instructions. MIC-values were read after 48 h for *G. vaginalis*, *Lactobacillus *spp. and *Bifidobacterium *spp. and after 72 h for *A. vaginae.*

## Results and discussion

### Value and reproducibility of Etest for MIC-determination of anaerobes

In this study we used the Etest for MIC-determination of vaginal bacteria towards 15 antibiotics. Croco *et al*. [25] concluded, from a study whereby the Etest was compared with the reference agar dilution method, that the Etest was a quantitatively accurate and reproducible method for routinely testing the antimicrobial susceptibilities of anaerobes, in particular of organisms associated with BV. Discordances were reported only for clindamycin susceptibility testing of lactobacilli and chloramphenicol susceptibility testing of the *Bacteroides fragilis *group. Schieven *et al*. [26] reported an agreement between the Etest and the reference agar dilution method within 1 log_2 _dilution of 87.4% for clindamycin and 85.1% for metronidazole for a total of 150 anaerobic isolates tested.

### Antimicrobial susceptibility testing of *Atopobium vaginae*

The antibiotic susceptibility for ampicillin, azithromycin, bacitracin, cefuroxime, ciprofloxacin, clindamycin, colistin, doxycycline, kanamycin, linezolid, metronidazole, nalidixic acid, penicillin, rifampicin and vancomycin obtained for nine *Atopobium vaginae *isolates in this study is listed in additional file 1 [see Additional file 1].

We focused our research on the antibiotic susceptibility profile of *Atopobium vaginae*. In contrast to the results of Ferris *et al*. [10] and Geißdörfer *et al*. [24], who reported MIC-values of more than 32 μg/ml for all four *A. vaginae *isolates tested (Table 1), we found a great variability in susceptibility for metronidazole, ranging from 2 to more than 256 μg/ml. When taking into account 16 μg/ml as the breakpoint between sensitive and resistant (NCCLS guidelines), only four out of 9 strains in our study could be accounted as intermediately susceptible or resistant. All nine *A. vaginae *isolates in our study showed MIC-values of more than 256 μg/ml for nalidixic acid and colistin while being inhibited by low concentrations of clindamycin (range: < 0.016 μg/ml), rifampicin (< 0.002 μg/ml), azithromycin (< 0.016 – 0.32 μg/ml), penicillin (0.008 – 0.25 μg/ml), ampicillin (< 0.016 – 0.94 μg/ml), ciprofloxacin (0.023 – 0.25 μg/ml) and linezolid (0.016 – 0.125 μg/ml) (Table 1).

**Table 1:** Comparison of MIC-values as determined for *Atopobium vaginae *by Ferris *et al*. [8] and Geißdörfer *et al*. [24] and in this study.

	**This study**		**Geißdörfer *et al*. [24]^a^**	**Ferris *et al*. [8]^b^**
**Number of strains**	**9**		**1**	**3**

**Antimicrobial agent**	**MIC_50_^c^**	**Range^c^**	**MIC^c^**	**Range^c^**

Ampicillin	0.023	< 0.016 – 0.94	0.032	
Ampicillin/sulbactam				0.06 – 0.25
Azitromycin	< 0.016	< 0.016 – 0.32		
Bacitracin	3	1 – 4		
Cefoxitin			2	2
Ceftriaxone				0.5 – 2
Cefuroxime	0.125	0.016 – 0.25	0.19	
Ciprofloxacin	0.064	0.023 – 0.25		
Clindamycin	< 0.016	< 0.016		< 0.015
Colistin	> 1024	> 1024		
Doxycyclin	0.38	0.19 – 0.75		
Imipenem			0.016	< 0.015 – 0.5
Kanamycin	12	8 – 16		
Linezolid	0.047	0.016 – 0.125		0.06 – 0.25
Meropenem				< 0.015 – 0.5
Metronidazole	8	2 - > 256	>16	> 32
Moxifloxacin				0.06 – 1
Nalidixic acid	> 256	> 256		
Penicillin	0.094	0.008 – 0.25	0.125	
Rifampicin	< 0.002	< 0.002		
Trovafloxacin				< 0.015 – 2
Vancomycin	1.5	1 – 4		

Legend

a. Tested with Etest and agar dilution method.

b. Tested with broth microdilution method for anaerobic bacteria with Brucella Broth supplemented with vitamin K (1 μg/ml), hemin (5 μg/ml) and laked horse blood (5%).

c. Data in μg/ml.

We previously described the existence of a second genotype within *A. vaginae*. To this genotype belongs isolate PB2003/189-T1-4, of which the 16S rRNA gene sequence differs at 23 positions compared to the type strain, but is identical to that of an isolate no longer in our collection of which the sequence was submitted [Genbank: AJ585206] [5]. This genotype 2 isolate showed a marked difference only for azithromycin (i.e. MIC of 0.32 μg/ml) with the other isolates (MIC of < 0.016 μg/ml).

### Antimicrobial susceptibility testing of *Gardnerella vaginalis*

We tested 4 strains of *Gardnerella vaginalis*. By using the Etest method in an anaerobic environment we found in general slightly lower values for ampicillin (range: < 0.016 – 0.047 μg/ml), penicillin (0.004 – 0.047 μg/ml), cefuroxim (< 0.016 – 0.125 μg/ml) and rifampicin (0.5 – 0.75 μg/ml) (Table 1), compared to other publications [25,27,28] [see Additional file 2]. The range of the MIC-values of *Gardnerella vaginalis *for clindamycin (range: < 0.016 – 0.047 μg/ml), colistin (> 1024 μg/ml), doxycycline (0.25 – 32 μg/ml), kanamycin (16 – 32 μg/ml), metronidazole (0.75 – 16 μg/ml), nalidixic acid (> 256 μg/ml) and vancomycin (0.125 – 0.38 μg/ml) is comparable with other studies [25,27-31] [see Additional file 2]. *G. vaginalis *is, according to NCCLS standards for anaerobic bacteria, susceptible to ampicillin (range: < 0.016 – 0.047 μg/ml), penicillin (0.004 – 0.047 μg/ml) and azithromycin (< 0.016 – 0.047 μg/ml). In 1993, Goldstein *et al*. [29] reported that 20% of *G. vaginalis *strains were resistant to metronidazole (MIC ≥ 16 μg/ml). In 2002, the same group reported a resistance of 29% to metronidazole for *G. vaginalis *[30]. All four strains in our study were susceptible.

### Antimicrobial susceptibility testing of *Lactobacillus *spp

Most of the *Lactobacillus *spp. we tested show a low MIC-value for ampicillin (range for all lactobacilli: 0.064 – 0.5 μg/ml), azithromycin (0.023 – 0.125 μg/ml), cefuroxim (< 0.016 – 1 μg/ml), linezolid (0.19 – 1.5 μg/ml), penicillin (0.047 – 0.19 μg/ml), rifampicin (0.016 – 2 μg/ml) and vancomycin (0.38 – 1 μg/ml) [see Additional file 1]. All strains were resistant to metronidazole (range for all lactobacilli: > 256 μg/ml). The strains of the species *L. crispatus*, *L. jensenii *and *L. ga*ss*eri *were all highly resistant to ciprofloxacin (range: > 32 μg/ml) in contrast to both *L. iners *strains (range: 0.25 – 0.38 μg/ml).

The single strain of *L. crispatus *tested yielded a very low MIC-value for clindamycin (< 0.016 μg/ml) and a high MIC-value for rifampicin compared to the other lactobacilli. The strain of *L. gasseri *tested showed comparable results to those of previous publications [32,33] for clindamycin, metronidazole, penicillin and vancomycin [see Additional file 2], indicating that this species is much more resistant to clindamycin than *L. crispatus*.

All lactobacilli were resistant to metronidazole (> 256 μg/ml) but susceptible for clindamycin (0.023 – 0.125 μg/ml). There were no literature data to compare the susceptibility of *L. iners *and *L. jensenii *for the antimicrobial agents tested in this study.

### Antimicrobial susceptibility testing of *Bifidobacterium *spp

Both of the tested *Bifidobacterium *strains are sensitive to a low amount of ampicillin (*B. longum*: 0.25 μg/ml, *B. breve*: 0.38 μg/ml), azithromycin (*B. longum*: 0.064 μg/ml, *B. breve*: 0.25 μg/ml), clindamycin (*B. breve *and *B. longum*: < 0.016 μg/ml), linezolid (*B. longum*: 0.25 μg/ml, *B. breve*: 0.38 μg/ml), penicillin (*B. longum*: 0.19 μg/ml, *B. breve*: 0.5 μg/ml), rifampicin (*B. longum*: 0.25 μg/ml, *B. breve*: 0.19 μg/ml) and vancomycin (*B. longum*: 0.38 μg/ml, *B. breve*: 1 μg/ml) [see Additional file 1]. The strains tested showed comparable results with previous publications except for linezolid and kanamycin [see Additional file 2], where we obtained a lower value than described [33-35].

## Conclusion

Bacterial vaginosis is a polymicrobial disease and the organisms involved are likely to be in symbiotic relationship to each other for various metabolic requirements. Antimicrobial treatment may affect susceptible members of the consortia which may negatively alter the microenvironment for resistant organisms, such as *A. vaginae *and *G. vaginalis*.

By knowing the antibiotic susceptibility of the vaginal species it might be possible to develop new regimens for the treatment of recurrent bacterial vaginosis.

For example, this study showed that metronidazole resistance of *A. vaginae *is not an intrinsic feature. Further research needs to make clear whether this metronidazole resistance might be acquired by the presence and activation of *nim*-genes [36]. Metronidazole resistance up to 29% [30] has been described for *G. vaginalis *but mechanisms are not yet clarified. Possibly this could be due to the lack of nitroreductases necessary to produce the hydroxymetabolite of metronidazole, which has stronger antibiotic activity than the parent compound.

Lactobacilli are resistant to metronidazole and it has been demonstrated that recolonistation of the vagina by H_2_O_2_-producing lactobacilli after metronidazole treatment occurs more frequently compared to clindamycin treatment [37].

Clindamycin, which is frequently used as a treatment for bacterial vaginosis, has indeed higher activity against *G. vaginalis *and *A. vaginae *than metronidazole, but *L. crispatus *is more susceptible to clindamycin than *L. gasseri *with as a consequence that a regimen of clindamycin can remove also the H_2_O_2_-producing lactobacilli from the vaginal microflora (Table 2). Hydrogen peroxide production is generally believed to be an important factor in the preservation of a normal vaginal microflora [1], i.e. in the build up of vaginal colonisation resistance. Therefore, it is possible that clindamycin treatment may diminish the vaginal colonisation resistance. Moreover, resistance to clindamycin seems to develop more readily than resistance to metronidazole, as becomes apparent from clinical studies comparing both antibiotics [23,37].

**Table 2:** Overview of the MIC-value ranges for *Atopobium vaginae *and *Gardnerella vaginalis *for 15 antimicrobial agents.

	***Atopobium vaginae *(n = 9)**	***Gardnerella vaginalis *(n = 4)**
**Antimicrobial agent**	**Range^a^**	**Range^a^**

Ampicillin	< 0.016 – 0.94	< 0.016 – 0.047
Azithromycin	< 0.016 – 0.32	< 0.016 – 0.047
Bacitracin	1 – 4	0.75 – 2
Cefuroxim	0.016 – 0.25	< 0.016 – 0.125
Ciprofloxacin	0.023 – 0.25	0.75 – 2
Clindamycin	< 0.016	< 0.016 – 0.047
Colistin	> 1024	> 1024
Doxycycline	0.19 – 0.75	0.25 – 32
Kanamycin	8 – 16	16 – 32
Linezolid	0.016 – 0.125	0.125 – 0.19
Metronidazole	2 – 256	0.75 – 16
Nalidixic acid	> 256	> 256
Penicillin	0.008 – 0.25	0.004 – 0.047
Rifampicin	< 0.002	0.5 – 0.75
Vancomycin	1 – 4	0.125 – 0.38

Legend

a. Data in μg/ml.

## Competing interests

The author(s) declare that they have no competing interests.

## Authors' contributions

EDB, RV, GC, GV and MV participated in the development of the study design, the analysis of the study samples, analysis and interpretation of the data, and in the writing of the report. HV and MT participated in the development of the study design, analysis and interpretation of the data, and in the writing of the report. All authors read and approved the final manuscript.

## Pre-publication history

The pre-publication history for this paper can be accessed here:



## Supplementary Material

Additional File 1 **Antibiotic susceptibility testing of vaginal bacteria for 15 antibiotics**. The table lists the antibiotic susceptibility for ampicillin, azithromycin, bacitracin, cefuroxime, ciprofloxacin, clindamycin, colistin, doxycycline, kanamycin, linezolid, metronidazole, nalidixic acid, penicillin, rifampicin and vancomycin obtained for nine strains of *Atopobium vaginae*, four strains of *Gardnerella vaginalis*, two strains of *Lactobacillus iners *and one strain each of *Bifidobacterium breve*, *B. longum, Lactobacillus crispatus, L. gasseri *and *L. jensenii*. Click here for file

Additional File 2 **Comparison between the results from our study and those from other publications**. The table lists the results from our study for the antibiotic susceptibility of *Bifidobacterium breve*, *B. longum*, *Gardnerella vaginalis*, *Lactobacillus crispatus *and *L. gasseri *compared to those those from other publications. Click here for file
