# Supplementary material for: Antibiotic susceptibility of Atopobium vaginae
Source: BMC Infect Dis. 2006 Mar 16;6:51. doi: 10.1186/1471-2334-6-51 (PMC1468414; doi:10.1186/1471-2334-6-51)
Supplement: Additional File 1 — Antibiotic susceptibility testing of vaginal bacteria for 15 antibiotics. The table lists the antibiotic susceptibility for ampicillin, azithromycin, bacitracin, cefuroxime, ciprofloxacin, clindamycin, colistin, doxycycline, kanamycin, linezolid, metronidazole, nalidixic acid, penicillin, rifampicin and vancomycin obtained for nine strains of Atopobium vaginae, four strains of Gardnerella vaginalis, two strains of Lactobacillus iners and one strain each of Bifidobacterium breve, B. longum, Lactobacillus crispatus, L. gasseri and L. jensenii. [file 1471-2334-6-51-S1.pdf]

Additional file 1: Antibiotic susceptibility testing of vaginal bacteria for 15 antibiotics.

Species	Reference number	AM[a]	AZ	BA	XM	CI	CM	CO	DC	KM	LZ	MZ	NA	PG	RI	VA
Atopobium vaginae	CCUG 42099	0.094	< 0.016	3	0.25	0.064	< 0.016	> 1024	0.75	12	0.094	2/2[b]	> 256	0.25	< 0.002	2.0
Atopobium vaginae	CCUG 44116	0.032	< 0.016	3	0.125	0.25	< 0.016	> 1024	0.19	12	0.032	4/4	> 256	0.064	< 0.002	1.5
Atopobium vaginae	CCUG 44258	0.023	< 0.016	4	0.19	0.064	< 0.016	> 1024	0.38	16	0.023	> 256/> 256	> 256	0.094	< 0.002	1.5
Atopobium vaginae	PB2003/009-T1-4	< 0.016	< 0.016	1	0.016	0.023	< 0.016	> 1024	0.38	16	0.125	12/12	> 256	0.008	< 0.002	1.0
Atopobium vaginae	PB2003/017-T1-2	< 0.016	< 0.016	1.5	0.125	0.032	< 0.016	> 1024	0.25	16	0.125	> 256	> 256	0.008	< 0.002	1.5
Atopobium vaginae	CCUG 38953[T]	0.094	< 0.016	3	0.023	0.032	< 0.016	> 1024	0.25	8	0,125	> 256/> 256	> 256	0.125	< 0.002	1.5
Atopobium vaginae	CCUG 44125	0.047	< 0.016	3	0.25	0.064	< 0.016	> 1024	0.19	12	0.047	8/8	> 256	0.19	< 0.002	1.0
Atopobium vaginae	CCUG 44061	0.023	< 0.016	3	0.19	0.047	< 0.016	> 1024	0.38	12	0.023	16/16	> 256	0.19	< 0.002	1.5
Atopobium vaginae	PB2003/189-T1-4	0.016	0.32	3	0.125	0.19	< 0.016	> 1024	0.38	16	0.016	6 / 8	> 256	0.008	< 0.002	1.0
Bifidobacterium breve	FB034-05AE	0.38	0.25	3	8	4	< 0.016	> 1024	24/32	> 256	0.38	> 256	> 256	0.5	0.19	1
Bifidobacterium longum	FB050-05AN	0.25	0.064	1	1.5	6	< 0.016	256	0.5	32	0.25	4	> 256	0.19	0.25	0.38
Gardnerella vaginalis	BVS 26	< 0.016	0.023	1	0.125	1	< 0.016	> 1024	0.5/0.25	32	0.25	0.75	> 256	0.016	0.5	0.38
Gardnerella vaginalis	FB022-1	< 0.016	< 0.016	0.75	< 0.016	1.5	< 0.016	> 1024	32/24	24	0.125	16	> 256	0.004	0.5	0.125
Gardnerella vaginalis	FB061-3	< 0.016	< 0.016	2	0.032	2	< 0.016	> 1024	0.75/0.5	16	0.25/0.125	1	> 256	0.008	0.75	0.5
Gardnerella vaginalis	FB049-2	0.047	0.047	2	0.094	0.75	0.032/0.047	> 1024	0.25	32	0.19	1	> 256	0.047	0.75	0.38
Lactobacillus crispatus	PB2003/023-T3-2	0.125	0.064	8	1	> 32	< 0.016	96	4	32	1.5	> 256	> 256	0.094	2	0.38
Lactobacillus jensenii	PB2003/035-T2-1	0.25	0.125	0.75	1	> 32	0.125	86	12	24	0.38	> 256	> 256	0.047	0.64	1
Lactobacillus gasseri	PB2003/051-T3-2. LAB114	0.125	0.094	48	1	> 32	4	> 1024	2	> 256	1.5	> 256	> 256	0.047	0.016	1
Lactobacillus iners	PB2003/195-T1-1	0.064	0.094	1.5	< 0.016	0.25	0.19	96	2	6	0.125	> 256	64	0.047	0.94	0.75
Lactobacillus iners	FB088-1	0.5/0.38	0.023	1	0.032	0.38	0.125	192	0.064	8	0.19	> 256	> 256	0.19	0.094	0.75

Legend:

AM: ampicillin; **AZ**: azithromycin; **BA**: bacitracin; **XM**: cefuroxime; **CI**: ciprofloxacin; **CM**: clindamycin; **CO**: colistin; **DC**: doxycycline; **KM**: kanamycin; **LZ**: linezolid; **MZ**: metronidazole; **NA**: nalidixic acid; **PG**: penicillin; **RI**: rifampicin; **VA**: vancomycin.

a. Data in µg/ml.

b. Values before and after slash indicate results from duplicate testing.
