# Supplementary material for: Antibiotic susceptibility of Atopobium vaginae
Source: BMC Infect Dis. 2006 Mar 16;6:51. doi: 10.1186/1471-2334-6-51 (PMC1468414; doi:10.1186/1471-2334-6-51)
Supplement: Additional File 2 — Comparison between the results from our study and those from other publications. The table lists the results from our study for the antibiotic susceptibility of Bifidobacterium breve, B. longum, Gardnerella vaginalis, Lactobacillus crispatus and L. gasseri compared to those those from other publications. [file 1471-2334-6-51-S2.pdf]

Additional file 2: Comparison between the results from our study and those from other publications.

Species[a]	Antimicrobial agent	This study	Other studies				Method[b]	Medium[c]	Incubation[d]	Reference
		Range[e]	MIC_{50}[e]	MIC_{90}[e]	Range[e]	Number of strains tested				
Bifidobacterium longum	Ampicillin	0.25	3.12	6.25	< 0.19 - 12.5	21	BDM	A	AN	[34]
	Clindamycin	< 0.016	≤ 0.5	64	≤ 0.5 - ≥ 256	47	Sensititre Anaero3 kit and ADM	B and C	AN	[33]
	Kanamycin	32	200	800	100 - 800	21	BDM	A	AN	[34]
	Linezolid	0.25	1	1	0.5 - 1	14	ADM	D	AN	[35]
	Metronidazole	4	2	≥ 64	≤ 0.5 - ≥ 64	47	Sensititre Anaero3 kit and ADM	B and C	AN	[33]
	Nalidixic acid	> 256	400	800	200-800	21	BDM	A	AN	[34]
	Penicillin G	0.19	0.12	0.25	0.12 - 2	47	Sensititre Anaero3 kit and ADM	B and C	AN	[33]
	Penicillin G	0.19	0.25	0.5	≤ 0.06 - 1	14	ADM	D	AN	[35]
	Penicillin G	0.19	0.39	0.78	< 0.19 - 0.78	21	BDM	A	AN	[34]
	Vancomycin	0.38	≤ 2	≤ 2	≤ 2 - 4	47	Sensititre Anaero3 kit and ADM	B and C	AN	[33]
	Vancomycin	0.38	0.5	1	0.25 - 1	14	ADM	D	AN	[35]
	Vancomycin	0.38	0.78	3.12	0.39 - 3.12	21	BDM	A	AN	[34]
Bifidobacterium breve	Linezolid	0.38	1	1	1	6	ADM	D	AN	[35]
	Penicillin G	0.5	0.25	0.5	≤ 0.06 - 0.5	6	ADM	D	AN	[35]
	Vancomycin	1	0.5	0.5	0.25 - 1	6	ADM	D	AN	[35]
Gardnerella vaginalis	Ampicillin	< 0.016 - 0.047	0.5	1	0.25 – 4	24	Etest	E	AN	[25]
	Ampicillin	< 0.016 - 0.047	0.5	0.5	0.03 – 1	93	ADM	F	AE	[27]
	Cefuroxime	< 0.016 - 0.125	1	4	0.06 – 4	93	ADM	F	AE	[27]
	Ciprofloxacin	0.75 - 2	1	2	1 – 4	93	ADM	F	AE	[27]
	Clindamycin	< 0.016 - 0.047	< 0.06	< 0.06	< 0.06	56	ADM	G	AN	[28]
	Clindamycin	< 0.016 - 0.047	0.5	1	≤ 0.12 - 1	24	Etest	E	AN	[25]
	Clindamycin	< 0.016 - 0.047	0.01	0.03	0.007 - 0.03	93	ADM	F	AE	[27]
	Clindamycin	< 0.016 - 0.047	0.03	0.03	0.03 - 0.06	15	ADM	D	AN	[29]
	Clindamycin	< 0.016 - 0.047	≤ 0.03	0.125	≤ 0.03 - 0.25	108	ADM	D	AN	[30]
	Colistin	> 1024	> 128	> 128	> 128	56	ADM	G	AN	[28]
	Doxycycline	0.25 - 32	1	16	0.125 - 32	108	ADM	D	AN	[30]
	Kanamycin	16 - 32	8	16	4 – 32	56	ADM	G	AN	[28]
	Metronidazole	0.75 - 16	< 0.03	> 16	< 0.03 - > 16	9	ADM	H	AE	[31]
	Metronidazole	0.75 - 16	8	16	2 – 128	93	ADM	F	AN	[27]
	Metronidazole	0.75 - 16	8	64	0.06 - 128	15	ADM	D	AN	[29]
	Metronidazole	0.75 - 16	8	> 32	≤ 0.25 - >32	108	ADM	D	AN	[30]

Species[a]	Antimicrobial agent	This study	Other studies	Method[b]	Medium[c]	Incubation[d]	Reference	Medium[c]	Incubation[d]	Reference
		Range[e]	MIC_{50}[e]	MIC_{90}[e]	Range[e]	Number of strains tested				
	Nalidixic acid	> 256	> 128	> 128	> 128	56	ADM	G	AN	[28]
	Penicillin G	0.004 - 0.047	0.035	0.07	0.035 – 0.15	56	ADM	G	AN	[28]
	Penicillin G	0.004 - 0.047	0.12	0.5	0.015 -0.5	93	ADM	F	AE	[27]
	Rifampicin	0.5 - 0.75	1	2	0.5-0.5[f]	93	ADM	F	AE	[27]
	Vancomycin	0.125 - 0.38	0.25	0.5	0.25 – 0.5	56	ADM	G	AN	[28]
	Vancomycin	0.125 - 0.38	0.25	0.5	0.12 – 0.5	93	ADM	F	AE	[27]
Lactobacillus crispatus	Clindamycin	< 0.016	0.12	> 256	≤ 0.03 - > 256	6	Etest	I	AN	[32]
Lactobacillus gasseri	Clindamycin	4	4	8	≤ 0.5 – 8	20	Sensititre Anaero3 kit and ADM	B and C	AN	[33]
	Clindamycin	4	16	32	8 – 32	4	Etest	I	AN	[32]
	Metronidazole	> 256	≥ 64	≥ 64	≥ 64	20	Sensititre Anaero3 kit and ADM	B and C	AN	[33]
	Penicillin G	0.047	≤ 0.06	0.12	≤ 0.06 – 0.12	20	Sensititre Anaero3 kit and ADM	B and C	AN	[33]
	Vancomycin	1	≤ 2	8	≤ 2 – 8	20	Sensititre Anaero3 kit and ADM	B and C	AN	[33]

Legend:

a. In this study one strain was tested for each species, except for *G. vaginalis* (4 strains).

b. ADM: Agar Dilution Method; BDM: Broth Dilution Method; Sensititre Anaero3 kit: Trek Diagnostic Systems, East Grinstead, UK.

c. Media: A: Tryptic Soy Broth, supplemented with 0.2% yeast extract and 0.06% L-cysteine.HCl; B: *Brucella* Standard Broth, supplemented with haemin and vitamin K1; C: MRS-cystein Agar; D: *Brucella* Agar, supplemented with 5% laked sheep blood and vitamin K1 (1 mg/L); E: *Brucella* Blood Agar; F: Human Blood Agar; G: Mueller-Hinton Agar, supplemented with 5% lysed horse blood; H: DST Agar; I: MRS Agar.

d. AN: anaerobic incubation; AE: aerobic incubation.

e. Data in µg/ml.

f. Data as published by the original authors.
